# Comparison of second-line therapy in IVIg-refractory Kawasaki disease: a systematic review

**DOI:** 10.1186/s12969-019-0380-z

**Published:** 2019-11-27

**Authors:** Courtney B. Crayne, Chace Mitchell, Timothy Beukelman

**Affiliations:** 1 Division of Pediatric Rheumatology, University of Alabama, 1600 7th Avenue S, CPPN G10, Birmingham, AL 35233 USA; 2 University of Alabama School of Medicine, 1670 University Blvd, Birmingham, AL 35233 USA

**Keywords:** IVIg-refractory Kawasaki disease, Infliximab, Coronary artery aneurysm

## Abstract

**Background:**

Evidence remains contradictory regarding second-line therapy in patients with Kawasaki disease (KD) refractory to initial intravenous immunoglobulin (IVIg). The objective of this study aims to evaluate the efficacy and safety of three treatments [i.e. a second IVIg infusion, methylprednisolone (IVMP), and infliximab (IFX)] in patients with refractory KD.

**Methods:**

A systematic search of PubMed, Embase, Cochrane, and ClinicalTrials.gov using predefined MeSH terms was performed from 1990 through 2017. Relevance screening was performed by two independent reviewers. Inclusion criteria included English-only, original clinical data. Eight studies met the inclusion criteria. Fever resolution, coronary lesions, and adverse event outcomes were extracted and pooled for analysis.

**Results:**

Of the 388 patients included from the 8 studies analyzed, a majority received a second IVIg dose (*n* = 263, 68%). Fever resolution was comparable between IVIg (72%) and IVMP (73%). IFX (88%) significantly increased fever resolution by approximately 20% compared to IVIg re-dose (RR 1.2; [95% CI: 1.1–1.4]; *p* = 0.03) and IVMP (RR 1.2; [95% CI: 1.0–1.5]; *p* = 0.04). Clinical significance of differences in coronary outcomes remains unclear.

**Conclusions:**

This combined analysis was limited due to variability in design and data reporting methods between the studies and risk of bias. In the absence of a clinical trial, IFX monotherapy as second-line treatment should be considered in patients who fail to respond to initial IVIg. This conclusion is based on a systematic review of the literature with pooled outcome data analysis suggesting IFX is more effective in fever resolution compared to a second IVIg dose and IVMP.

## Background

Characterized by fever and mucocutaneous features, Kawasaki disease (KD) is an acute, self-limited medium vessel vasculitis most commonly affecting infants and young children < 5 years of age [1, 2]. KD is markedly more prevalent in Japan with an annual incidence of 243–265 per 100,000 children compared to 20–25 per 100,000 in the US [3]. Complete KD requires persistent fever ≥5 days plus 4 out of 5 clinical criteria including bilateral nonexudative bulbar conjunctivitis, polymorphous nonvesicular rash, oropharyngeal changes, unilateral cervical lymphadenopathy, and swelling of extremities followed by desquamation [2].

Coronary artery aneurysms are a well-recognized complication of KD, occurring in roughly 25% of untreated disease and the leading cause of acquired heart disease in developed countries [4]. If administration of intravenous immunoglobulin (IVIg) is given during the first 10 days of fever, the risk of coronary abnormalities during the first 30 days is reduced from about 25% with aspirin alone to approximately 5% [5]. Two classification criteria exist for diagnosis of coronary artery dilation and aneurysms. The Japanese Ministry of Health (JHM) criteria classify coronary arteries using absolute or relative internal lumen diameter. Dilation is defined as an internal lumen diameter > 3 mm in children < 5 years old or > 4 mm in children ≥5 years old or if the internal diameter of a segment measures ≥1.5 times that of an adjacent segment [6]. JMH criteria is more commonly used in Japan and given the variability in lumen sizes with respect to body size, may underestimate the incidence of coronary artery dilations and aneurysms. The 2004 American Heart Association (AHA) adjusts for body surface area (BSA) and classifies solely on Z-scores. Per AHA criteria, dilation is defined as a Z-score ≥ 2 and < 2.5 and aneurysms are diagnosed if Z-score is ≥2.5. Dilation often resolves within 4–8 weeks after fever onset. Giant aneurysms, defined as ≥8 mm per JHM and AHA or Z-score ≥ 10 per AHA criteria, are unlikely to regress [1, 4, 5, 7].

Approximately 10–20% of patients fail to respond to IVIg and remain febrile ≥36 h following completion of the IVIg infusion and are thus classified as IVIg-refractory or IVIg-resistant [4]. Persistent fever is reported to increase the risk of coronary lesions by as high as nine-fold compared to children who responded to the initial IVIg [8]. At present, there are no strong recommendations regarding second-line therapy in IVIg-refractory KD. The AHA 2017 Scientific Statement recommends one of three most common second-line therapies: a second IVIg dose of 2 g/kg; intravenous methylprednisolone (IVMP) 30 mg/kg for 3 days with or without an oral glucocorticoid taper; or a single infliximab (IFX) infusion of 5 mg/kg [4]. There are no adequately powered studies examining the response rates or the effects on coronary artery lesions to second-line therapy, and as such, there is no consensus on the preferred second-line agent in children refractory to initial IVIg.

The objective of this systematic review was to evaluate the efficacy and safety of the three most common second-line monotherapies (i.e. a second IVIg infusion, IVMP, and IFX) in patients with IVIg-refractory KD using a meta-analysis approach, hypothesizing that alternative treatment will be more effective than retreatment with a second IVIg dose in patients who fail to respond to the initial IVIg dose.

## Methods

A systematic review of the literature was conducted by two independent reviewers (i.e., CC and CM) using the Preferred Reporting Items for Systematic Reviews and Meta-Analyses (PRISMA) checklist. There was no discordance between reviewers.

### Data sources and search

Using predefined MeSH terms in 4 databases, a search of PubMed, Embase, Cochrane Library, and ClinicalTrials.gov was performed for studies published from January 1, 1990 until November 17, 2017. In PubMed the following search terms were used: “Mucocutaneous Lymph Node Syndrome”[Mesh] OR (Kawasaki* [tiab] AND (syndrome [tiab] OR disease [tiab])) OR “Mucocutaneous Lymph Node Syndrome” AND Refract* [tiab] OR “Drug Resistance”[Mesh] OR resistant [tiab] OR resistance [tiab] OR unresponsiv* [tiab] OR nonresponsiv* [tiab] OR non-responsiv* [tiab] OR “Retreatment”[Mesh] OR adjunct* [tiab] AND “Drug Therapy”[Mesh] OR “drug therapy” [Subheading] OR therapy [tiab] OR therapies [tiab] OR therapeutic [tiab] OR treat [tiab] OR treating [tiab] OR treated [tiab] OR treatment* [tiab] OR “therapeutic use” [Subheading] OR “Therapeutics”[Mesh] OR “Adrenal Cortex Hormones”[Mesh] OR corticosteroid* [tiab] OR “Infliximab”[Mesh] OR Remicade [tiab] OR Avakine [tiab] OR flixabi [tiab] OR inflectra [tiab] OR infliximab [tiab] OR remsima [tiab] OR revellex [tiab] OR steroid [tiab] OR steroids [tiab] OR “Steroids”[Mesh]. Similar search strings were used in the other databases. A manual search was completed after the original search and prior to manuscript preparation with one additional study deemed relevant. No authors were contacted. Articles were limited to the English language. No review protocol exists for this study.

### Study selection criteria

Predefined criteria were applied to assess study eligibility. The population was restricted to children ages 0 months to 18 years with KD refractory to initial IVIg (2 g/kg) who remained febrile > 38 °C 36 h after completion of initial IVIg and who received second-line monotherapy with either a second IVIg dose (2 g/kg), IVMP (30 mg/kg/dose × 3 days), or IFX (5–7 mg/kg × 1 dose). The outcomes of interest included fever response, coronary artery lesions, hospitalization duration, and adverse events. Only original research was included. Study design was restricted to peer-reviewed full-text publications with at least 5 patients. Prospective and retrospective studies were included, and studies could be either observational, randomized or not randomized (i.e. open label). Studies written in a language other than English, duplicate data, abstract proceedings and reviews, basic science studies, surgical or procedural outcomes, combination therapy, alternate dosing of initial IVIg or second-line therapy, multiple retreatments, and case reports and series < 5 patients were excluded from analysis.

### Outcomes of interest

Refractory was defined as persistent fever (i.e. ≥ 38 °C) > 36 h to < 7 days after initial IVIg (2 g/kg) completion. The primary outcome measure was fever resolution within 36 h of completion of second-line therapy. Independent of fever response, incidence and size of coronary artery lesions per JHM criteria prior to second-line monotherapy and at 4–8 weeks following fever onset was extracted. Secondary outcomes included fever duration, time to fever resolution, hospitalization duration, and adverse events. Adverse events were categorized as serious or non-serious using the United States Food and Drug Administration (FDA) classification. Serious adverse events resulted in death or near-death, prolonged hospitalization, disability, intervention to prevent permanent impairment, or any medical event that resulted in additional medical treatment to prevent another serious event.

### Data extraction

Of the studies meeting inclusion criteria, data abstraction included year of publication, country of origin, study characteristics, second-line treatment, number of subjects, fever response outcomes, coronary artery lesions prior to second-line therapy and at 4–8 week follow-up based on JHM and AHA criteria, number of giant aneurysms, adverse events, hospitalization duration, total fever duration, and time to fever resolution. Risk of bias was assessed using the Cochrane Methods [9].

### Statistical analyses

Data were combined and grouped by second-line therapy. Because studies meeting inclusion criteria were not restricted to comparative studies, we first summarized the proportion of observed outcomes for each treatment using a random effects model. To account for low variance in some studies (e.g., 100% response rates), a meta-analysis of proportions using the Freeman-Tukey double arcsine transformation was performed. There were an insufficient number of studies identified for infliximab as second-line treatmentin order to perform meta-regression. Instead, because the meta-analysis proportions and the crude proportions were similar, we calculated the incidence rate ratios of the crude proportions to estimate their relative differences and determined a 95% confidence interval. A *p*-value of less than 0.05 was considered significant. Stata 14 (StataCorp; College Station, TX, USA) statistical software was used for analysis.

## Results

**Fig. 1:**
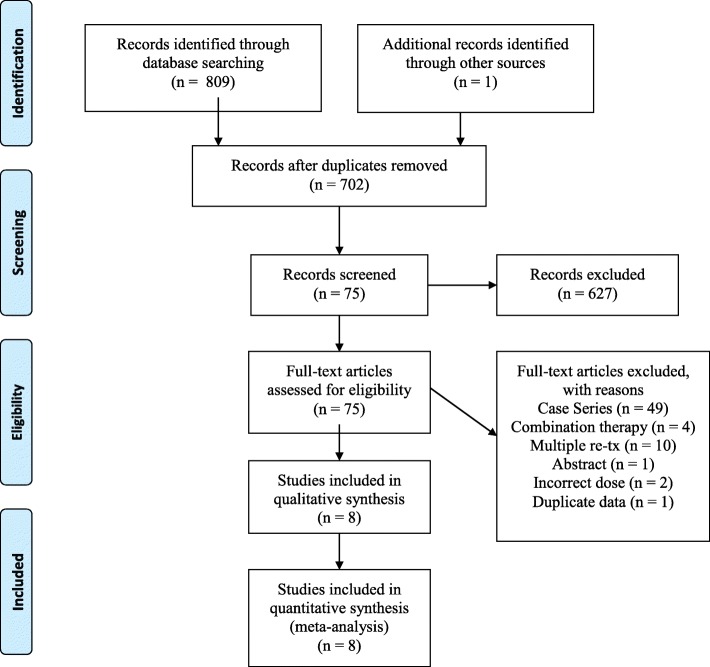
PRISMA flow diagram of study inclusion

A total of 810 potentially relevant publications were identified. After title-abstract screening, 75 full-text publications were reviewed, resulting in 8 relevant studies for inclusion in the pooled cohort analysis (Fig. 1) [10–17]. Miura et al. (2005) defined fever as ≥37.5 °C at > 48 h after initial IVIg. The fever resolution responses were similar between treatment groups. This study was included for side effect profile only. Miura et al. (2011) reported a third treatment with IVMP in patients refractory to the second IVIg dose. Outcomes to the third-line therapy were excluded, and only the second-line monotherapy data was included in the systematic review analysis. Additionally, Singh et al. reported a cohort treated with infliximab for variable diagnoses. Only patients treated with infliximab as second-line therapy for IVIg-refractory KD were included in the systematic review analysis. All of the patients received an initial IVIg (2 g/kg) and high dose ASA (range 30–100 mg/kg) and remained refractory with persistent fever. All patients received second-line monotherapy with a second dose of IVIg (2 g/kg), IVMP (30 mg/kg/day × 3 days), or IFX (5–7 mg/kg × 1 dose). Five studies originated in Japan. The remaining three studies were from the US, Korea, and India (Table 1).

**Table 1:** Study characteristics

Study	Year	Country	Total (*N* = 388)	Study Characteristics		Tx A (n)	Tx B (n)	Aspirin dose per day
				Study Design	Randomization			
Son et al.	2011	US	106	Retrospective	N/A	IVIg (86)	IFX (20)	80-100 mg/kg until afebrile then 3-5 mg/kg
Youn et al.	2016	Korea	43	Prospective	+	IVIg (32)	IFX (11)	80-100 mg/kg until afebrile
Teraguchi et al.	2013	Japan	41	Prospective	+	IVIg (27)	IVMP (14)	30 mg/kg
Furukawa et al.	2008	Japan	63	Prospective	–	IVIg (19)	IVMP (44)	30 mg/kg tapered to 10 mg/kg then 5 mg/kg
Ogata et al.	2008	Japan	27	Retrospective	N/A	IVIg (14)	IVMP (13)	80-100 mg/kg
Miura et al.	2005	Japan	22	Prospective	+	IVIg (11)	IVMP (11)	
Miura et al.	2011	Japan	74	Prospective	–	IVIg (74)	–	30 mg/kg until afebrile then 5 mg/kg until 8 weeks
Singh et al.	2015	India	12	Retrospective	N/A	IFX (12)	–	30 mg/kg until afebrile then 3–5 mg/kg

### Risk of Bias assessment

Overall, the methodological quality of the studies meeting inclusion criteria was poor. Only three studies were prospective, randomized two-arm trials. None of these three studies were blinded. Two studies were prospective without randomization, and there were three retrospective studies (Tables 1, 2 and 3). With the exception of one study [10], baseline characteristics across treatment groups within each study were comparable. One study [11] was halted prematurely due to side effects from IVMP. Results across the studies had inconsistent reporting methods.

**Table 2:** Primary Outcomes by Study

	Study	Fever Resolution	Baseline Coronary Lesions	Persistent non giant lesions	Giant coronary lesions
2nd IVIg (2 g/kg)	Son et al. ^a^	65/86 (76%)	NR ^a^	NR ^a^	NR ^a^
	Youn et al.	21/32 (66%)	0/32 (0%)	4/32 (13%)	0/32 (0%)
	Teraguchi et al.	21/27 (78%)	7/27 (26%	4/27 (15%)	0/27 (0%)
	Furukawa et al.	12/19 (63%)	2/19 (11%)	0/19 (0%)	0/19 (0%)
	Ogata et al.	14/14 (100%)	2/14 (14%)	0/14 (0%)	1/14 (7%)
	Miura et al. (2005)	5/11 (45%)	NR	NR	NR
	Miura et al. (2011)	53/74 (72%)	NR	2/74 (3%)	0/74 (0%)
	Total	191/263 (73%, [95% CI: 67–78%])	11/60 (18%, [95% CI: 10–30%])	10/166 (6%, [95% CI: 3–11%])	1/166 (1%, [95% CI: 0–3%])
	Meta-analysis proportion	74% (95% CI: 63–83%)*	10% (95% CI: 0–28%)*	5% (95% CI: 0–12%)	0% (95% CI: 0–1%)
IVMP (30 mg/kg/d × 3 d)	Teraguchi et al.	7/14 (50%)	5/14 (36%)	3/14 (21%)	1/14 (7%)
	Furukawa et al.	34/44 (77%)	5/44 (11%)	2/44 (5%)	3/44 (7%)
	Ogata et al.	13/13 (100%)	0/13 (0%)	0/13 (0%)	0/13 (0%)
	Miura et al. (2005)	5/11 (45%)	NR	NR	NR
	Total	59/82 (72%, [95% CI: 61–81%])	10/58 (17%, [95% CI: 9–29%])	5/71 (7%, [95% CI: 2–16%])	4/71 (6%, [95% CI: 2–14%])
	Meta-analysis proportion	73% (95% CI: 45–94%)*	12% (95% CI: 0–34%)*	6% (95% CI: 0–19%)	4% (95% CI: 0–12%)
IFX (5–7 mg/kg ×1)	Son et al. ^Ŧ^	17/20 (85%)	NR ^Ŧ^	NR ^Ŧ^	NR ^Ŧ^
	Youn et al.	10/11 (91%)	0/11 (0%)	1/11 (9%)	0/11 (0%)
	Singh et al.	11/12 (92%)	5/12 (42%)	1/12 (8%)	0/12 (0%)
	Total	38/43 (88%, [95% CI: 75–96%])	5/23 (22%, [95% CI: 8–24%])	2/23 (9%, [95% CI: 1–28%])	0/23 (0%, [95% CI: 0–15%])
	Meta-analysis proportion	89% (95% CI: 76–97%)	15% (95% CI: 2–34%)*	9% (95% CI: 0–25%)	0% (95% CI: 0–8%)
Combined Total		288/388 (74%, [95% CI: 70–79%])	26/141 (18%, [95% CI: 12–26%])	17/260 (7%, [95% CI: 4–10%])	17/260 (2%, [95% CI: 1–4%])

*IVIg* intravenous immunoglobulin, *IVMP* intravenous methylprednisolone, *IFX* infliximab, *NR* not reported

^a^ 29 total (34% giant) CALs (reported using AHA critieria and median z-score); Ŧ 7 (35% giant) CALs (reported using AHA critieria and median z-score); * test for heterogeneity *p* < 0.05

**Table 3:** Comparison of fever response and coronary artery lesions between treatments

	Fever Response		Persistent non-giant dilation		Giant Aneurysm	
Comparison	Risk Ratio [95% CI]	*P* value	Risk Ratio [95% CI]	*P* value	Risk Ratio [95% CI]	*P* value
IVIg vs IVMP	1.0 [0.9–1.2]	0.9	0.9 [0.3–2.6]	0.9	0.1 [0.01–0.9]	0.01*
IFX vs IVMP	1.2 [1.0–1.5]	0.04*	1.2 [0.3–5.9]	0.8	0	0.6^#^
IFX vs IVIg	1.2 [1.1–1.4]	0.03*	1.3 [0.3–5.6]	0.7	0	1^#^

*IVMP* intravenous methylprednisolone, *IFX* infliximab

*p < 0.05; #one-sided Fischer’s exact test

### Fever response outcomes

Of the 388 subjects included in this study, 263 (68%) received a second IVIg infusion, 82 (21%) received IVMP, and 43 (11%) received IFX. Overall, 74% [95% CI, 70–79%] of patients receiving any of the three treatments had a resolution of fever (Table 2). Fever resolution was comparable in those receiving a second IVIg infusion (73%; [95% CI, 67–78%]) and in those receiving IVMP (72%; 95% CI: 61–81%]) (RR = 1.0; [95% CI: 0.9–1.2]; *p* = 0.9). Infliximab (88%; [95% CI: 75–96%]) increased fever resolution by approximately 20% compared to a second IVIg dose (RR 1.2; [95% CI: 1.1–1.4]; *p* = 0.03) and IVMP (RR 1.2; [95% CI: 1.0–1.5]; *p* = 0.04) (Tables 2 and 3).

### Coronary artery outcomes

There were no significant differences in aneurysm presence at baseline prior to each 2nd line therapy. The incidence of baseline aneurysms was 18% in the second IVIg group, 17% in the IVMP group, and 22% in the IFX group. There were also no significant differences in persistent non-giant aneurysms at 4–8 weeks following 2nd line therapy across the three treatment groups. IVIg (6%) compared to IVMP (7%) was associated with a risk ratio of 0.9 [95% CI, 0.3–2.6] (*p* = 0.9), and IVIg compared to IFX (9%) was associated with a risk ratio of 1.3 [95% CI, 0.3–5.6] (*p* = 0.7). IFX compared to IVMP was associated with a risk ratio of 1.2 [95% CI, 0.3–5.9] (*p* = 0.8) (Tables 2 and 3).

IVIg (1%) significantly reduced the presence of giant aneurysms by 90% versus IVMP (6%) (RR = 0.1; [95% CI, 0.01–0.9], *p* = 0.01) (Tables 2 and 3). There were zero giant aneurysms observed following second-line treatment with IFX, but data were only available for 23 patients (Tables 2 and 3). The coronary artery outcomes from Son et al. were reported as median Z–scores using the AHA criteria and were therefore excluded from the combined analysis. Using a Z-score cutoff of > 3, which includes small to giant aneurysms, there was no statistically significant difference in coronary artery aneurysms at 6 weeks following a second IVIg dose (35%) compared to IFX (34%) [14].

### Adverse events

A higher proportion of non-serious adverse events were reported in patients receiving IVMP (24%; [95% CI, 16–35%]). These included electrolyte and serum glucose abnormalities, hypertension, hypothermia, bradycardia, and one GI bleed. There was one report of transient fibular nerve paralysis, classified as a serious event, in a patient who received IVMP [10]. Infusion reactions and transient hepatomegaly were more common in patients receiving IFX (16%; [95% CI 7–31%]) compared to a second IVIg (4%; [95% CI 2–7%]). There was one death of unknown cause reported 2 months following diagnosis in a patient who failed to respond to a second IVIg infusion and subsequently received IVMP and IFX [14] (Table 4).

**Table 4:** Secondary Outcomes by Study

	Study	Hospitalization Duration	Fever Duration	Days to fever resolution	NonSerious Adverse Events	Serious Adverse Events	Death
2nd IVIg (2 g/kg) median (range)	Son et al.	6 (2–20)	10 (5–37)	NR	3/86 (3%)	1/86 (1%)	1/86 (1%)
	Youn et al.	10 (8–12)	total NR	0.71 (0.17–2.6)	5/32 (16%)	0/32 (0%)	0/32 (0%)
	Teraguchi et al.	NR	10 (6–14)	1 (1–3)	0/27 (0%)	0/27 (0%)	0/27 (0%)
	Furukawa et al.	NR	total NR	NR	0/19 (0%)	0/19 (0%)	0/19 (0%)
	Ogata et al.	mean 12 ± 2.1	mean 11 ± 2	mean 3 ± 2.4	0/14 (0%)	0/14 (0%)	0/14 (0%)
	Miura et al. (2005)	NR	NR	NR	2/11 (18%)	0/11 (0%)	0/11 (0%)
	Miura et al. (2011)	NR	NR	NR	0/74 (0%)	0/74 (0%)	0/74 (0%)
	Total	–	–	–	10/263 (4%, [95% CI: 2–7%])	1/263 (0.4%, [95% CI: 0–2%])	1/263 (0.4%, [95% CI: 0–2%])
IVMP (30 mg/kg/d × 3 d) median (range)	Teraguchi et al.	NR	9.5 (7–18)	< 24 h	1/14 (7%)	0/14 (0%)	0/14 (0%)
	Furukawa et al.	NR	total NR	NR	11/44 (25%)	1/44 (2%)	0/44 (0%)
	Ogata et al.	mean 14.5 ± 2	mean 8 ± 2.1	mean 1 ± 1.3	2/13 (15%)	0/13 (0%)	0/13 (0%)
	Miura et al. (2005)	NR	NR	NR	6/11 (55%)	0/11 (0%)	0/11 (0%)
	Total	–	–	–	20/82 (24%, [95% CI: 16–35%])	1/82 (1%, [95% CI: 0–7%])	0/82 (0%, [95% CI: 0–4%])
IFX (5–7 mg/kg ×1) median (range)	Son et al.	5.5 (4–35)	8 (5–14)	NR	6/20 (30%)	0/20 (0%)	0/20 (0%)
	Youn et al.	8 (7–9)	total NR	0.25 (0.08–1)	1/11 (9%)	0/11 (0%)	0/11 (0%)
	Singh et al.	NR	NR	NR	0/12 (0%)	0/12 (0%)	0/12 (0%)
		–	–	–	7/43 (16%, [95% CI: 7–31%])	0/43 (0%, [95% CI: 0–8%])	0/43 (0%, [95% CI: 0–8%])

*IVMP* intravenous methylprednisolone, *IFX* infliximab, *NR* not reported

### Hospitalization duration

Hospitalization duration was only reported in 3 studies. Son et al. reported a median hospitalization duration of 6 days with a second IVIg and 5.5 days with IFX (*p* = 0.04). Both groups had comparable time from fever onset to diagnosis and both groups received second-line therapy 2 days after initial IVIg. Youn et al. reported a median hospital stay of 10 days in patients receiving a second IVIg and 8 days in patients receiving IFX (*p* = 0.046) with no reference to the timing of second-line therapy. Ogata et al. reported a mean hospital stay of 12 ± 2.1 days with a second IVIg and 14.5 ± 2 days with IVMP, noting no significant difference. Neither of these two studies referenced the time of second-line therapy with respect to fever onset or initial IVIg (Table 4).

### Fever duration and time to resolution

Fever duration was also only reported in 3 studies. Son et al. reported a median fever duration of 8 days in the IFX group compared to 10 days following a second IVIg. Following a multivariate analysis controlling for age, platelet count, hemoglobin levels, and days from fever onset, this corresponded to 1.2 fewer days of fever in patients treated with IFX (*p* = 0.03). Teraguchi et al. reported a median fever duration of 10 days following a second IVIg and 9.5 days following IVMP (*p* > 0.05). There was no significant difference between the groups regarding the day of illness at initial IVIg or at second treatment. Ogata et al. reported a significant reduction in fever duration among patients receiving IVMP (mean 8 ± 2.1) compared to a second IVIg (mean 11 ± 2) (*p* < 0.05). There was no significant difference between the mean day of illness at the time of second treatment (7 days and 8 days, respectively). There was no reference to the day of illness at time of initial IVIg (Table 4).

Days to fever resolution following second line therapy were also reported in only 3 studies. Youn et al. reported a median fever resolution time of 6 h following IFX compared to 17 h following a second IVIg (*p* = 0.042). Ogata et al. reported a mean response time of 1 ± 1.3 days following IVMP and 3 ± 2.4 days following a second IVIg (*p* < 0.05). Teraguchi et al. reported a median fever resolution of 1 day following a second IVIg and within 24 h following IVMP (Table 4).

## Discussion

The results of this systematic review of the literature revealed that in published reports, the majority of children with KD who fail to respond to the initial IVIg and remained febrile received a second IVIg infusion. Combined analysis of the reported study results, however, suggest that IFX may be more effective in reducing fever compared to a second IVIg and IVMP. Controlling for several confounders, Son et al. found that IFX resulted in 1.2 fewer days of fever which corresponded to 0.5 fewer days of hospitalization [14]. Overall, IFX may result in a 20% increase in fever resolution response compared to IVIg retreatment and IVMP if given as second-line monotherapy in IVIg-refractory KD. The results of this systematic review differ from Chan et al. meta-analysis which found that both IFX and IVMP were more effective than a second IVIg dose due to the antipyretic effects. They found no difference in cardiac outcomes between the three groups. In comparison, the meta-analysis included combination therapy with IVIg plus IVMP in addition to monotherapy. Seven of the studies included in this study were also included in the Chan et al. meta-analysis. The differences in results are likely due to the variations in methodology [18].

Infliximab is a chimeric monoclonal antibody against tumor necrosis factor (TNF). Inhibition of TNF has anti-inflammatory effects and has been used to treat vasculitic diseases [19, 20]. Serum TNF levels are elevated in patients with KD and have been associated with IVIg failure and increased risk for coronary artery aneurysms [21–24]. Persistent fever following initial IVIg in KD may increase the risk of coronary artery lesions up to nine-fold [8]. IFX may lower the risk of adverse coronary events through cytokine blockade as evidenced by the fever resolution.

Interpretation of coronary artery lesion outcomes using the combined cohort was limited. Comparison of the three treatment groups suggests no apparent difference in non-giant coronary artery lesions at baseline or at 4–8 weeks following fever resolution. The use of the JMH criteria likely underestimated the incidence of lesions. There were no reported giant aneurysms in the IFX group, but data were available for only 23 of these patients, making interpretation limited. When given in combination with IVIg as initial therapy, IFX did not reduce treatment resistance or the frequency of adverse coronary events [25]. Son et al. reported coronary lesions using Z-scores > 3 per AHA and due to the design of this review restricting the criteria to JHM, these results were excluded from the combined analysis [14], but no overall difference between the treatments in development of any size aneurysm was suggested. This study included all aneurysms, including small with Z-score > 3. Comparison of giant aneurysms between the IVIg retreatment group and the IVMP showed a statistically significant difference; however, the wide confidence interval makes clinical significance uninterpretable.

IVMP was associated with more non-serious non-life-threatening adverse events compared to IFX and IVIg. Infusion reactions were more common in patients receiving IVIg and IFX. There was one death in a non-responder to a second IVIg dose. This patient also received IVMP and IFX as third- and fourth-line therapies [14].

This systematic review and combined analysis has several limitations, notably the large variability between the studies and the high risk of bias. Likewise, there were substantially fewer patients who received IFX (11%) compared to a second IVIg (68%), and of the IFX group, approximately 75% was retrospective. Prospective, randomized trials are necessary in determining the risk of coronary artery lesions in patients who remain febrile following initial IVIg. It is unclear if there is an associated risk of worsening coronary lesions in patients who failed initial IVIg therapy but responded to second-line therapy with fever resolution compared to patients who failed both initial and second-line therapy and remained febrile. Further, it remains unclear if the risk of coronary artery aneurysms varies with respect to second-line treatment.

IVIg-refractory KD is rare, making an adequately powered prospective, randomized control trial (RCT) difficult to conduct. The results of this target review suggest that IFX may be a more effective monotherapy in reducing fever in IVIg-refractory disease compared to a second IVIg dose or IVMP, and this may in turn reduce the hospitalization duration. There is a prospective, randomized trial currently enrolling to compare a second IVIg dose to IFX (ClinicalTrials.gov Identifier: NCT02298062).

## Conclusion

In the absence of randomized control trial data, in patients who fail to respond to initial IVIg and remain febrile, IFX monotherapy should be considered as an effective second-line treatment for fever resolution. This conclusion is based on a systematic review of the literature with pooled outcome data analysis from 8 studies suggesting IFX is more effective in fever resolution compared to a second IVIg dose and IVMP. Clinical significance of coronary artery sequelae remains unclear.

## Data Availability

Not applicable, systematic review.

## Abbreviations

AHA: American Heart Association
BSA: Body surface area
CAL: Coronary artery lesions
CI: Confidence interval
IFX: Infliximab
IVIg: Intravenous immunoglobulin
IVMP: Intravenous methylprednisolone
JHM: Japanese Ministry of Health
KD: Kawasaki disease
